# A revision of the Neotropical genus *Paraberismyia* Woodley (Diptera, Stratiomyidae, Beridinae) with three new species

**DOI:** 10.3897/zookeys.353.6301

**Published:** 2013-11-20

**Authors:** Norman E. Woodley

**Affiliations:** 1 Systematic Entomology Laboratory-PSI-ARS-USDA, Smithsonian Institution NHB-168, P. O. Box 37012, Washington, DC 20013-7012, USA

**Keywords:** Beridinae, Mexico, Neotropical Region, new species, taxonomy

## Abstract

The Neotropical genus *Paraberismyia* Woodley, 1995, is revised. Three new species, *P. chiapas* **sp. n.**, *P. mathisi* **sp. n.**, and *P. triunfo* **sp. n.** are described, all having type localities in Chiapas, Mexico. A key to the four known species is provided.

## Introduction

Woodley (1995) described the genus *Paraberismyia* based on a single new species, *Paraberismyia tzontehuitza* Woodley, in the stratiomyid subfamily Beridinae. At the time that the genus was described, a few specimens representing several new species were known. It was hoped that additional material would become available, but since then no new specimens have been collected. The purpose of this revision is to describe the new species that are currently known.

*Paraberismyia* is known only from the state of Chiapas in southern Mexico and Totonicapán Department in southwestern Guatemala. Specimens with associated altitudinal data have been collected at a minimum of 1300 meters and range to more than 2800 meters. Despite some recent collecting in areas further south in Central America, particularly several extensive surveys at all elevations in Costa Rica, *Paraberismyia* has not been found south of Guatemala.

## Methods

Specimens have been utilized from two institutions for which acronyms are given that are used in the specimen data citations:

CNC Canadian National Collection of Insects, Agriculture and Agri-Food Canada, Ottawa, Ontario, Canada

USNM Department of Entomology, National Museum of Natural History, Smithsonian Institution, Washington, DC, USA

Specimens were examined with a Zeiss Stemi SV 11 stereomicroscope. Male terminalia were dissected from specimens relaxed in a humidity chamber for about 24 hours, cleared in hot KOH, neutralized with weak acetic acid, and rinsed with water. The terminalia are preserved in a microvial on the specimen pin. Morphological terminology follows that of McAlpine (1981) as modified by Cumming and Wood (2009). Body lengths are given exclusive of antennae.

## Taxonomy

### Paraberismyia

Woodley, 1995

http://species-id.net/wiki/Paraberismyia

Paraberismyia Woodley 1995: 134. Type species, *Paraberismyia tzontehuitza* Woodley, by original designation.

#### Diagnosis.

Woodley (1995) fully diagnosed and described *Paraberismyia* within the context of the world fauna of Beridinae. It may be separated from all other world genera of beridines except *Allognosta* Osten Sacken and *Berismyia* Giglio-Tos by having distinct hair-like setae around the apical margin of the first antennal flagellomere. *Paraberismyia* may be separated from *Allognosta* because it possesses denticles on the posterior margin of the scutellum, which *Allognosta* lacks. *Allognosta* is also not known to occur in Mexico or Central America. Species of *Berismyia* overlap in distribution with *Paraberismyia*. *Berismyia* are drab colored flies with a brownish to black mesonotum with at most faint metallic reflections and a dark brownish to black, unicolorous abdomen, whereas *Paraberismyia* are more brightly colored with a distinctly metallic mesonotum and an abdomen that is bicolored. Also, all known species of *Berismyia* (including several that are undescribed) have the genital capsule with a very long, narrow, posteromedial process on the synsternite that is sharply acute apically (Woodley 1995: 191, fig. 126), while in *Paraberismyia* this process is shorter, broader, and has a rounded apex (Figs 11, 20, 29).

#### Remarks.

The species of *Paraberismyia* are very similar in general structure, differing mainly in characters of coloration and the male terminalia. The coloration differences are very consistent and therefore can be used to accurately identify the species.

#### Key to species of Paraberismyia

**Table:** 

1	Males (eyes contiguous on frons)	2
–	Females (eyes widely separated on frons)	4
2(1)	Wing cell *cu*p completely covered with microtrichia; at least distal half of hind basitarsus dark brown	3
–	Wing cell *cu*p not completely covered with microtrichia, extensively bare in basal half; hind basitarsus completely pale colored, at most with vague darkening of extreme apex	*Paraberismyia triunfo* sp. n.
3(2)	Dorsal half of anepisternum with bare, shiny area without tomentum; hind basitarsus completely dark brown, only vaguely pale at extreme base (Fig. 27); wing cell r_2+3_ infuscated, essentially concolorous with cell r_4_	*Paraberismyia tzontehuitza* Woodley
–	Dorsal half of anepisternum completely covered with tomentum; hind basitarsus with basal one-third to one-half pale whitish yellow (Fig. 9); wing cell r_2+3_ weakly infuscated, obviously paler than cell r_4_	*Paraberismyia mathisi* sp. n.
4(1)	Wing cell *cu*p completely covered with microtrichia; pleura yellowish to orangish, not obviously marked with darker coloration (Figs 10, 28)	5
–	Wing cell *cu*p not completely covered with microtrichia, extensively bare in basal half; pleura with obvious dark markings or nearly completely dark (Figs 2, 19)	6
5(4)	Lateral margins of upper frons bare, without tomentum (Fig. 6); dorsal part of occiput (postocular orbit) only narrowly visible in profile with brownish gray tomentum that is not conspicuous (Fig. 28); anepisternum on dorsal half with bare, shiny area	*Paraberismyia tzontehuitza* Woodley
–	Lateral margins of upper frons with bands of grayish tomentum along eye margins (Fig. 4); dorsal part of occiput (postocular orbit) wide, easily visible in profile with conspicuous silvery gray tomentum (Fig. 10); anepisternum on dorsal half completely covered with tomentum	*Paraberismyia mathisi* sp. n.
6(4)	Postalar callus, postpronotal lobe, and laterotergite yellowish orange; abdominal sternites 2–5 completely yellowish orange	*Paraberismyia chiapas* sp. n.
–	Postalar callus and postpronotal lobe with at least some dark brownish coloration, laterotergite black with bluish reflections; abdominal sternites 2–5 with distinct dark lateral markings	*Paraberismyia triunfo* sp. n.

### Paraberismyia chiapas sp. n.

http://zoobank.org/9426518C-4A9D-4772-B395-4A1E763C2318

http://species-id.net/wiki/Paraberismyia_chiapas

Figs 1 –3

#### Diagnosis.

*Paraberismyia chiapas* can be distinguished from other species in the genus by the combination of having cell *cu*p only partially covered with microtrichia and all of the abdominal sternites completely yellowish orange. The other species with cell *cu*p only partly covered with microtrichia, *Paraberismyia triunfo*, has distinct dark markings on the sternites.

#### Description.

**Male.** Unknown.

**Figures 1–2.**
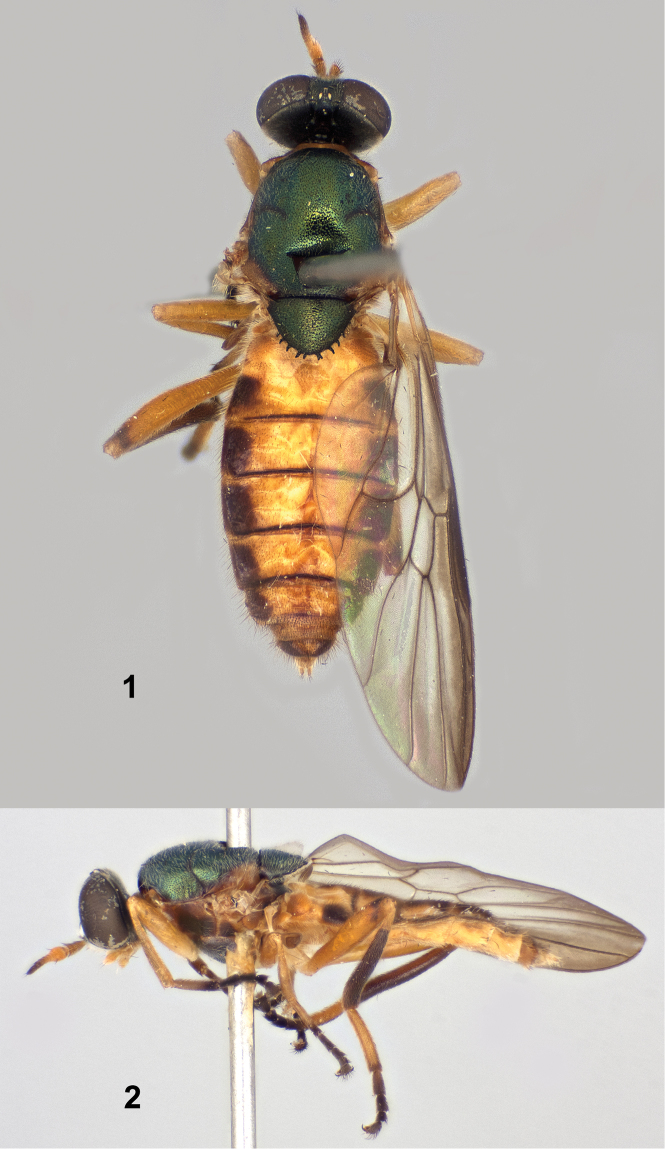
Holotype female of *Paraberismyia chiapas* Woodley. **1** Dorsal view **2** Left lateral view.

**Figures 3–6.**
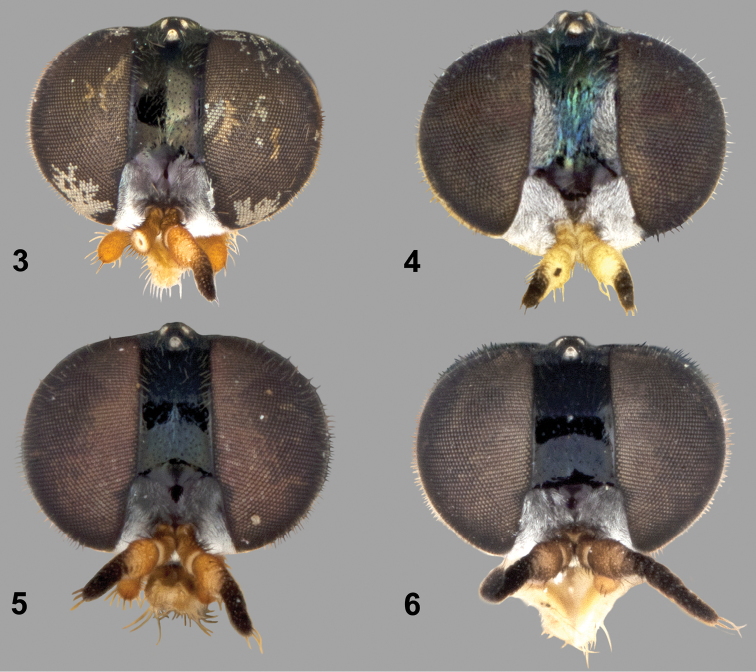
Frontal view of female heads of *Paraberismyia* species. **3** *Paraberismyia chiapas* Woodley (holotype)**4** *Paraberismyia mathisi* Woodley (paratype)**5** *Paraberismyia triunfo* Woodley (paratype) **6** *Paraberismyia tzontehuitza* Woodley (paratype).

**Female** (Figs 1, 2). *Head*: Black, without metallic reflections except upper frons has very faint greenish reflections (Figs 1, 3); upper frons 0.25 width of head at anterior ocellus; upper frons very finely punctate; lower frons and face densely grayish white tomentose, lower frons with medial, rounded bare area which extends from upper frons, occiput also tomentose except for median occipital sclerite, but tomentum is darker, brownish gray; upper frons with short, sparse pale hairs about one-half length of scape; face with pale hairs about two-thirds length of scape; gena with pale yellowish hairs a little longer than those of face, occiput with pale hairs becoming progressively shorter above gena; eye densely pilose, hairs pale, less than half length of scape; antenna 1.10 times length of head; first two segments and flagellomeres 1–5 yellowish, apical 3 flagellomeres brownish; first two antennal segments with stiff black hairs, longer hairs on flagellum black; palpus yellow, with numerous long hairs, most of which are pale yellowish, a few dark hairs present at apex of second segment; proboscis yellow.

*Thorax*: Scutum and scutellum dark metallic green (Fig. 1), postpronotal lobe and postalar callus dark yellow; pleura yellowish orange with ventral two-thirds of anepisternum, entire katepisternum and anterior two-thirds of anepisternum, and posterior half of meron brown to brownish black (Fig. 2) and subscutellum and mediotergite similarly colored; scutum and scutellum finely, densely punctate; thorax with inconspicuous pale tomentum present on prothorax, anepimeron, meron, subscutellum and mediotergite, difficult to observe; anepisternum on dorsal half bare and shiny medially; mostly pilose with more or less erect pale hairs, those on scutum and scutellum slightly appressed, about length of pedicel on dorsum, ranging to length of scape + pedicel on pleura with middle of anepisternum, entire katepimeron, meron, mediotergite, and subscutellum bare; legs (Fig. 2) yellowish, but hind femur with brownish area on extreme dorsoapical region, front tibia vaguely suffused with brownish color, hind tibia dark brownish except of extreme base and indistinct area ventrally on proximal half, front and middle tarsi are brownish-black except for basal two-thirds of basitarsi, although yellowish coloration is somewhat obscured by dark pilosity, hind tarsus brownish black but basitarsus is wholly yellow; legs short pilose, posterior surfaces of middle and hind femora with sparse, scattered longer hairs, posteroventral surface of hind tibia with a few longer hairs, coloration of pilosity generally similar to cuticular ground color, except basitarsi have some darker hairs on pale regions; wing hyaline with moderate brownish infuscation on anterior one-third and apex, veins brownish, yellowish at extreme base of wing; wing entirely covered with microtrichia except cell *cu*p is bare except at base and apex; halter yellowish, knob vaguely suffused with brownish.

*Abdomen*: Tergites brownish, except first tergite and central two-thirds of tergites 2–6 dark yellow with narrow extensions of yellow reaching lateral margin at anterior corners of tergites 3–5 (Fig. 1); tergite 7 similar but less distinctly marked, tergal grooves on tergites 2–5 brownish black; sternites entirely dark yellow except sternite 8 vaguely infuscated with brown; tergites vaguely, almost imperceptibly tomentose, quite shiny; pilosity of tergites mostly brownish and very short, some pale hairs on tergites 1 and 2, pilosity longer on lateral margins and wholly pale on tergite 1 and intermixed on tergites 2–4; sternites with short, yellowish hairs, a few dark hairs laterally on sternite 6, sternite 7 with pilosity completely brownish; cerci dark yellow, second segment ovoid and slightly shorter than first, with pale yellow hairs on both segments but dark hairs present at apex of second segment.

*Length*: 7.8 mm.

#### Distribution.

Known only from the state of Chiapas, Mexico.

#### Type material.

Holotype female (USNM), **MEXICO:** Chiapas, El Triunfo (49 km S of Jaltenango, 15°39.4'N, 92°48.5'W), 2000 meters, 13–15.v.1985, Amnon Freidberg. The holotype is missing the right antennal flagellum and the left wing, but is otherwise in good condition.

#### Etymology.

The species epithet, *chiapas*, is a noun in apposition based on the state of Chiapas, Mexico, where the type locality is located.

#### Remarks.

Although general coloration is difficult to characterize, in appearance this species has a more greenish mesonotum, and the yellow coloration is a little more orangish than in the other species.

### Paraberismyia mathisi sp. n.

http://zoobank.org/41597C79-F222-4FD8-9EB1-6D2A422158BA

http://species-id.net/wiki/Paraberismyia_mathisi

Figs 4 , 7 – 15

#### Diagnosis.

*Paraberismyia mathisi* can be distinguished from other species in the genus by having the dorsal half of the anepisternum completely covered with fine tomentum, without a bare, shiny medial area. No other species has this character state. Females have tomentose spots on the lateral margins of the upper frons (Fig. 4) and a relatively broad strip of the occiput (postocular orbit) visible behind the eye in profile covered in conspicuous grayish tomentum (Fig. 10). These two character states are also unique within the genus.

#### Description.

**Figures 7–8.**
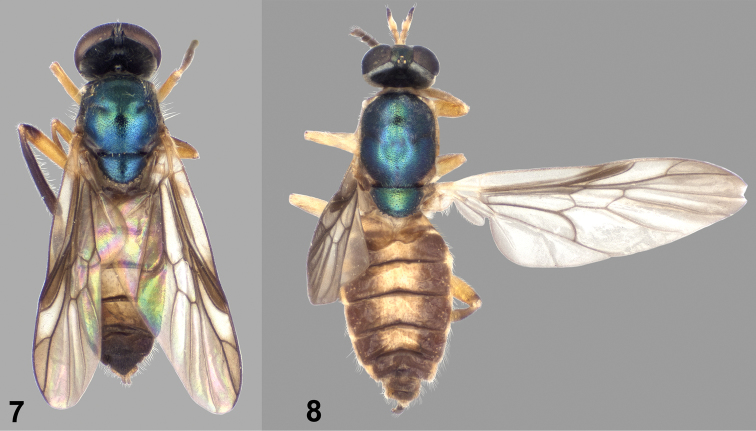
Dorsal views of *Paraberismyia mathisi* Woodley. **7** Male (paratype) **8** Female (paratype).

**Figures 9–10.**
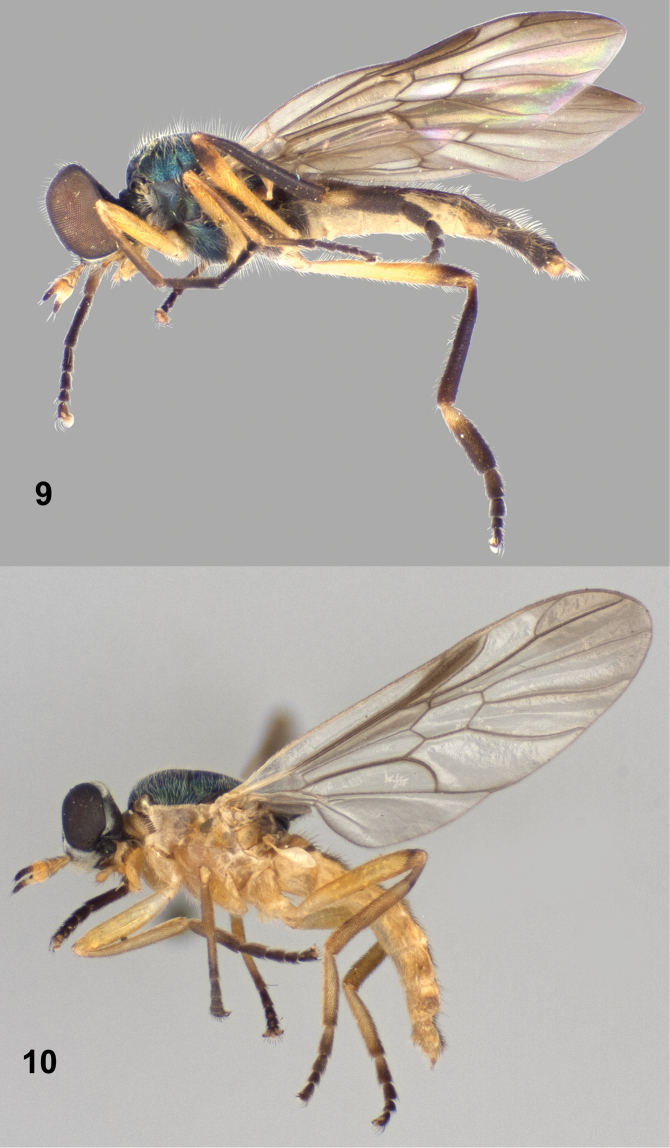
Left lateral views of *Paraberismyia mathisi* Woodley. **9** Male (paratype) **10** Female (paratype).

**Male** (Figs 7, 9). *Head*: Black, without metallic reflections; lower frons and face densely silvery gray tomentose, occiput also tomentose except for median occipital sclerite, but tomentum is sparser and dark, not strongly contrasting with background coloration, median area of upper frons very sparsely tomentose; upper frons with sparse dark hairs, lower frons just above antennae with mostly pale pilosity that is shorter than the first antennal segment; face pilose with mostly dark, longer hairs, with a few yellowish hairs intermixed; gena with pale yellowish hairs a little longer than those of face, occiput with scattered long pale hairs; eye densely pilose, hairs brownish black, about half length of first antennal segment; antenna 0.75–0.85 length of head, first two segments and first flagellomere yellowish, following two flagellomeres yellow internally, apical flagellomeres brownish black; first two antennal segments with stiff black hairs, longer hairs on flagellum black; palpus yellow, with numerous long hairs, those on first segment pale yellowish, second segment with hairs mostly black; proboscis yellow.

*Thorax*: Scutum and scutellum metallic bluish green (Fig. 7), postpronotal lobe and postalar callus brownish; pleura blackish brown, anepisternum and katepisternum with metallic reflections similar to coloration of mesonotum, posterior margin of anepimeron slightly yellowish; mesonotum finely, densely punctate; thorax with grayish tomentum present over most of prothorax, anterior two-thirds of anepisternum, entire katepimeron and meron, anatergite, mediotergite, and subscutellum, most conspicuous on anepisternum, which has the dorsal half completely tomentose medially; mostly pilose with long, erect pale hairs, a little longer than first two antennal segments combined, intermixed with short, pale, semi-appressed hairs on scutum and scutellum, with small strip on anterior part of anepisternum, entire katepimeron, meron, anatergite, mediotergite, and subscutellum bare; hind tarsus with tarsomeres 1–3 moderately inflated; legs (Fig. 9) yellowish, except hind femur brown on apical one-fifth, front tibia brownish anteriorly and dorsally, middle tibia with irregular pale brown infuscation, hind tibia entirely brownish black, and all tarsi are brownish-black except middle basitarsus is paler on basal half, and hind basitarsus is yellowish on basal one-fourth to one-half; legs short pilose, posterior surfaces of all femora with longer, erect pale hairs, posteroventral surface of hind tibia with scattered longer, erect hairs, coloration of pilosity similar to cuticular ground color, except narrow apices of femora have blackish hairs; wing hyaline with brownish infuscation anteriorly and apically, but noticeably hyaline in cells c and r_2+3_, cell r_1_ brown, veins brownish, yellowish at extreme base of wing; cell *cu*p with entire surface covered with microtrichia; halter yellowish, knob slightly darker than stem.

*Abdomen*: Tergites (Fig. 7) dark brownish with extensive translucent pale area on tergites 2–5, the pale area not discreetly defined, tergal grooves brown, tergite 6 and beyond brown; sternite 1 brown except for wide posterior margin pale yellowish, sternites 2–5 pale yellowish, 4 and 5 with vague lateral brown areas, sternite 6 and beyond brown; tergites vaguely, almost imperceptibly tomentose, quite shiny, with short, blackish pilosity, lateral margins with a fringe of pale hairs longer than antennal flagellum, tergites 5 and beyond with some dark hairs intermixed; sternites with short, yellowish hairs, becoming dark on dark-colored posterior segments.

**Figures 11–15.**
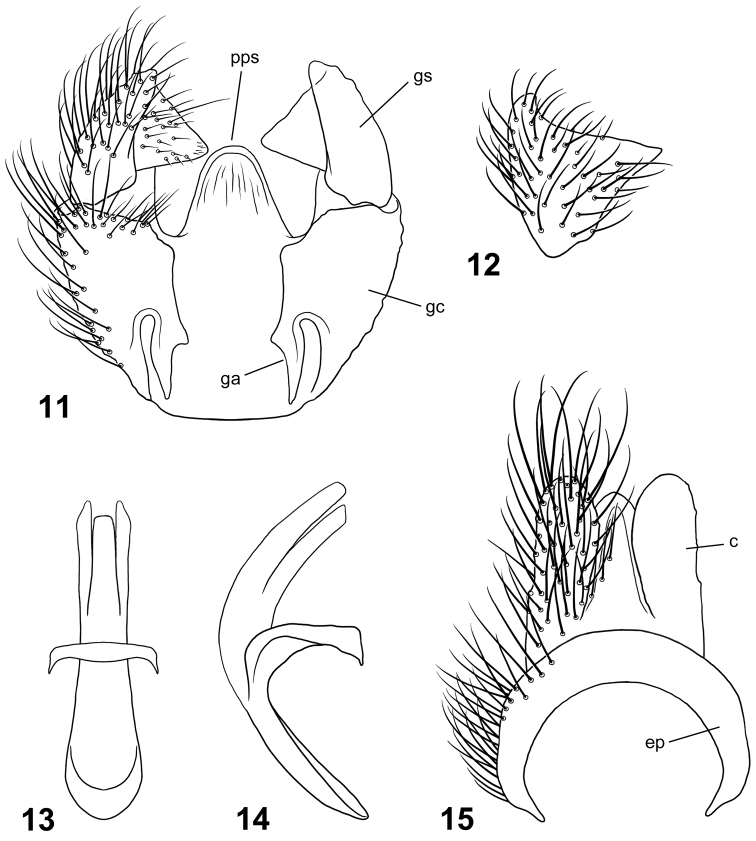
Male terminalia of *Paraberismyia mathisi* Woodley. **11** Genital capsule, dorsal view **12** Gonostylus, lateral view **13** Phallic complex, dorsal view **14** Phallic complex, left lateral view **15** Epandrium and postgenital segments. Abbreviations: *c*, circus; *ep*, epandrium; *ga*, gonocoxal apodeme; *gc*, gonocoxite; *gs*, gonostylus; *pps*, posteromedial process of synsternite.

*Terminalia*: Gonocoxites (Fig. 11) with lateral margins tapering anteriorly, slightly arcuate, with low, broadly rounded process ventral to gonostylus; gonocoxal apodemes short, not reaching anterior margin of genital capsule; synsternite of genital capsule with triangular-shaped process that is broadly rounded at apex (Fig. 11); gonostylus (Figs 11, 12) slightly arcuate, shorter than in *Paraberismyia tzontehuitza*, with internal triangular process near apex of ventral margin that is proportionately larger than in *Paraberismyia tzontehuitza*; phallic complex (Figs 13, 14) trifid, moderately arcuate in lateral view, lateral lobes nearly parallel, medial lobe only slightly shorter than lateral lobes; epandrium (Fig. 15) narrow, posterior margin evenly rounded; cercus of moderate width, apex moderately rounded.

*Length*: 5.7–6.1 mm.

**Female** (Figs 8, 10). Differs from male as follows: *Head*: Frons 0.25–0.28 width of head at anterior ocellus; upper frons (Fig. 4) black with greenish metallic reflections, very finely punctate, with lateral silvery gray tomentose spots, lower frons with inverted triangular bare area which extends from upper frons; occiput (posterior orbit) (Fig. 10) visible in profile, slightly wider than length of scape, densely silvery gray tomentose; pilosity of head shorter than in male, at most one-half length of scape, except on gena; upper frons evenly, sparsely pilose with pale hairs; antenna longer than in male, 0.96–1.04 length of head, flagellomeres 1–3 yellow; palpus more robust, entirely pale pilose except for a few dark hairs at apex.

*Thorax*: Scutum and scutellum metallic bluish green (Fig. 8), but postpronotal lobe yellow, postalar callus pale brownish, and entire remainder of thorax yellow (Fig. 10), except subscutellum and mediotergite, which are brownish; tomentum as in male, but more whitish on pale cuticular areas; pilosity generally shorter, on scutum at most as long as first antennal segment and without longer, erect hairs; hind tarsus without inflated tarsomeres; leg coloration as in male, but hind femur with a brownish black blotch near apex which is less extensive than in male, front tibia light brownish anteriorly, middle tibiae wholly yellow, hind tibia yellowish to brownish, not as dark as in male, middle basitarsus yellowish on basal half, hind basitarsus yellow except at apex; pilosity of legs similar to that of male, but posterior hairs on femora are shorter; halter pale yellowish.

*Abdomen*: Tergites (Fig. 8) with yellowish areas not especially translucent, this coloration confined to medial third of tergites 2–5; sternites entirely yellow; cerci small but robust, second segment shorter than first, first segment yellow, second slightly infused with brownish color, hairs yellowish except some on second segment dark.

*Length*: 5.4–5.6 mm.

#### Distribution.

Known only from the state of Chiapas, Mexico.

#### Type material.

Holotype male (USNM), **MEXICO:** Chiapas, El Triunfo (49 km S of Jaltenango, 15°39.4'N, 92°48.5'W), 1300–2000 meters, 13–15.v.1985, W.N. Mathis. The holotype is in excellent condition. Paratypes (all in USNM): 1 male, 2 females, same data as holotype; 2 males, same data as holotype except collected by A. Freidberg; 2 females, same data as holotype except elevation 1500 meters and collected by A. Freidberg; 1 male, same data as holotype except elevation 2000 meters and collected by A. Freidberg.

#### Etymology.

The species epithet, *mathisi*, is a patronym honoring Wayne N. Mathis of the Smithsonian Institution, who collected part of the type series. Wayne has been a colleague and friend for nearly 40 years. He has collected many interesting Stratiomyidae in the course of his extensive field work.

#### Remarks.

This distinctive species is the smallest in the genus, and has several character states not found in other species of the genus as noted in the diagnosis.

### Paraberismyia triunfo sp. n.

http://zoobank.org/1E28150D-6BED-4C78-A7F0-389E016C4A6E

http://species-id.net/wiki/Paraberismyia_triunfo

Figs 5 , 16 – 24

#### Diagnosis.

*Paraberismyia triunfo* can be distinguished from other species in the genus by having the combination of wing cell *cu*p not completely covered with microtrichia and abdominal sternites 2–5 with distinct dark lateral markings. It is also the only species in which the pleura of the female (Fig. 19) is completely dark, without yellow coloration.

#### Description.

**Figures 16–17.**
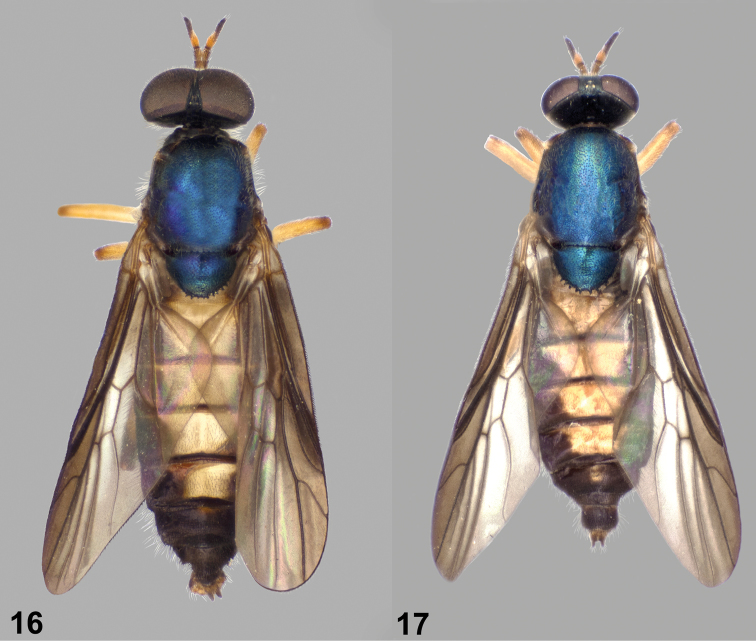
Dorsal views of *Paraberismyia triunfo* Woodley. **16** Male (paratype) **17** Female (paratype).

**Figures 18–19.**
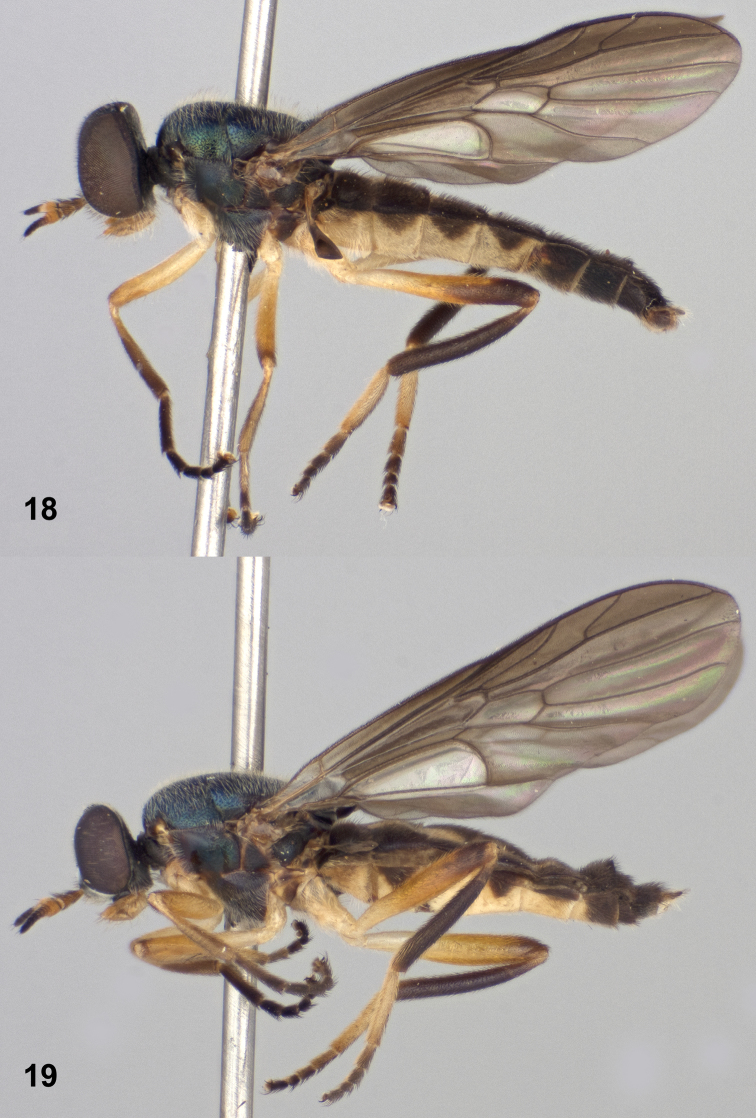
Left lateral views of *Paraberismyia triunfo* Woodley. **18** Male (paratype) **19** Female (paratype).

**Male** (Figs 16, 18). *Head*: Black, without metallic reflections; lower frons and face densely silvery gray tomentose, lower frons usually with a very narrow bare median line, occiput also tomentose except for median occipital sclerite, but tomentum is sparser and dark, not strongly contrasting with background coloration, median area of upper frons very sparsely tomentose; upper frons with scattered dark pilosity, lower frons just above antennae with brownish pilosity that is shorter than scape; face pilose with dark, longer hairs about length of scape, sometimes a few pale hairs intermixed; gena with pale yellowish hairs a little longer than those of face, occiput with scattered long pale hairs; eye densely pilose, hairs brownish black, about half length of scape; antenna 0.97–1.07 length of head, first two segments and first two flagellomeres yellowish, third and sometimes fourth flagellomeres yellow internally, apical flagellomeres brownish black; first two antennal segments with stiff black hairs, longer hairs on flagellum black; palpus dark yellow, with numerous long hairs, those on first segment pale yellowish, second segment with hairs mostly black; proboscis yellow.

*Thorax*: Scutum and scutellum metallic bluish (Fig. 16), but medial portion of scutum postsuturally and sometimes lateral margins of scutum bronzy black, postpronotal lobe and postalar callus brownish; pleura blackish brown, anepisternum and katepisternum with some dull greenish metallic reflections, posterior margin of anepimeron usually brownish; mesonotum finely, densely punctate; thorax with grayish tomentum present over most of prothorax, narrow anterior margin of anepisternum, entire katepimeron and meron, anatergite, much sparser on mediotergite, and dense and conspicuous on subscutellum; anepisternum on dorsal half bare and shiny medially; mostly pilose with long, erect pale hairs, a little longer than first two antennal segments combined, intermixed with short, pale, semi-appressed hairs on scutum and scutellum, with anteromedial part of anepisternum, entire katepimeron, meron, anatergite, mediotergite, and subscutellum without pilosity; hind tarsus with tarsomeres 1–3 weakly inflated; legs (Fig. 18) yellowish, except fore and mid femora can be weakly browned apically, hind femur brown on apical one-fourth to one-half, front tibia usually partly suffused with brownish coloration, hind tibia entirely brownish black, and all tarsi are brownish-black except middle basitarsus is paler on basal three-fourths, and hind basitarsus is yellowish, sometimes vaguely brownish at extreme apex dorsally; legs short pilose, posterior surfaces of all femora with longer, erect pale hairs, posteroventral surface of hind tibia with scattered longer, erect hairs, coloration of pilosity similar to cuticular ground color, except hind basitarsus, fore and mid tibiae, and apices of femora have blackish hairs; wing hyaline, evenly infuscate anteriorly and apically, weakly so on posterior part of wing, cell r_1_ brown, veins brownish, yellowish at extreme base of wing; cell *cu*p with with microtrichia but a large area medially is bare; halter with stem yellowish, knob dark brown.

*Abdomen*: Tergites (Fig. 16) dark brownish with extensive medial, translucent pale area on tergites 1–5, wider anteriorly on tergites 3–5, tergal grooves brown, tergite 6 and beyond brown; sternites 1–5 pale yellowish, sternites 2–5 with distinct lateral triangular brown markings, sternite 6 and beyond brown; tergites vaguely, almost imperceptibly tomentose, quite shiny, with short, blackish pilosity, lateral margins with a fringe of dark hairs longer than antennal flagellum; sternites with short, yellowish hairs, mostly dark on lateral markings and becoming dark on dark-colored posterior segments.

**Figures 20–24.**
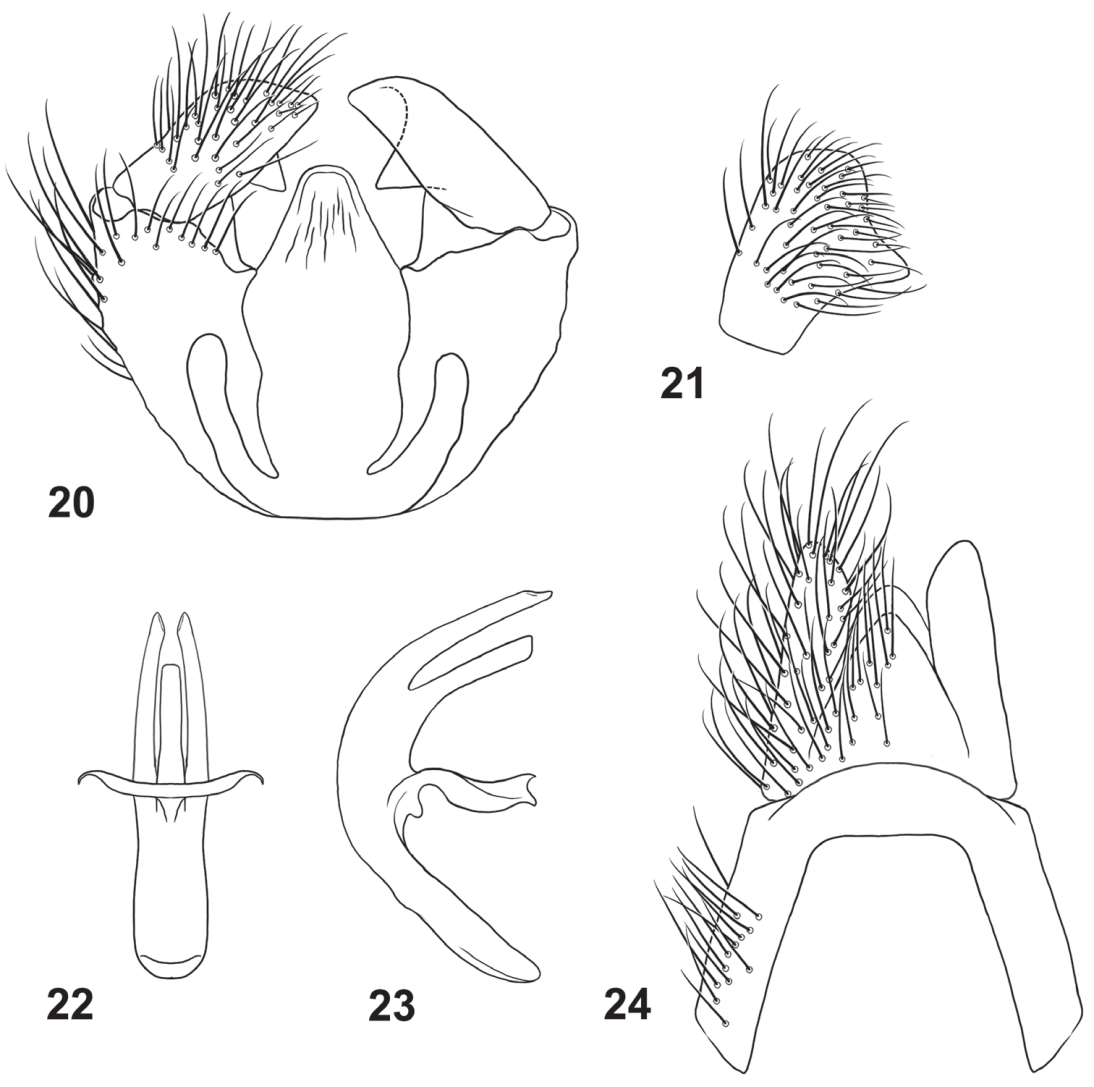
Male terminalia of *Paraberismyia triunfo* Woodley. **20** Genital capsule, dorsal view **21** Gonostylus, lateral view **22** Phallic complex, dorsal view **23** Phallic complex, left lateral view **24** Epandrium and postgenital segments.

*Terminalia*: Gonocoxites (Fig. 20) with lateral margins tapering anteriorly, arcuate, with low, broadly rounded process ventral to gonostylus; gonocoxal apodemes short, not reaching anterior margin of genital capsule; synsternite of genital capsule with triangular-shaped process that is moderately rounded at apex (Fig. 20); gonostylus (Figs 20, 21) slightly arcuate, shorter than in *Paraberismyia tzontehuitza*, with internal ventral triangular process on ventral margin that is more medial and proportionately smaller than in *Paraberismyia tzontehuitza*; phallic complex (Figs 22, 23) trifid, strongly arcuate in lateral view, lobes slender, lateral lobes very slightly arcuate medially, medial lobe shorter than lateral lobes; epandrium (Fig. 24) narrow, posterior margin evenly rounded but with angular corners; cercus of moderate width, apex moderately rounded.

*Length*: 7.2–7.5 mm.

**Female** (Figs 17, 19). Differs from male as follows: *Head*: Frons 0.20–0.23 width of head at anterior ocellus; upper frons (Fig. 5) black, without metallic reflections, very finely punctate; lower frons with inverted triangular bare area which extends from upper frons that has curved lateral margins; pilosity of head shorter than in male, at most one-half length of scape, except on gena; upper frons evenly, sparsely pilose with pale hairs; pilosity of face mostly to entirely pale; antenna longer than in male, 1.16–1.30 length of head; palpus more robust.

*Thorax*: Scutum and scutellum (Fig. 17) dark metallic greenish blue, without prescutellar bronzy area, but postpronotal lobe dark yellow around margins; pilosity generally shorter, on scutum at most as long as first antennal segment and without noticeable longer, erect hairs; hind tarsus (Fig. 19) without inflated tarsomeres; pilosity of legs similar to that of male, but posterior hairs on femora are shorter.

*Abdomen*: Tergites (Fig. 17) with yellowish areas not especially translucent; sternite 6 variably yellow along anterior margin and/or medially; cerci small but robust, second segment shorter than first, both segments brownish, pilosity dark.

*Length*: 6.3–7.3 mm.

#### Distribution.

Known only from the state of Chiapas, Mexico and Totonicapán Department in southwestern Guatemala.

#### Type material.

Holotype male (USNM), **MEXICO:** Chiapas, El Triunfo (49 km S of Jaltenango, 15°39.4'N, 92°48.5'W), 1300–2000 meters, 13–15.v.1985, W.N. Mathis. The holotype is in excellent condition. Paratypes (all in USNM): 5 males, 13 females, same data as holotype; 2 males, 2 females, same data as holotype except elevation 1500 meters and collected by A. Freidberg; 5 females, same data as holotype except elevation 2000 meters and collected by A. Freidberg; 3 males, **GUATEMALA:** Totonicapán Department, 14°55'N, 91°22'W, July 1902, Dr. Eisen.

#### Etymology.

The species epithet, *triunfo*, is a noun in apposition based on the name of the type locality.

#### Remarks.

The specimens from Guatemala were labeled “Totonicapán”, so it is uncertain if this refers to the department or the city within the department. I have included general geographic coordinates for the department in the specimen data citations above. The Guatemala specimens have a slightly paler overall appearance, but this is likely due to the age of the specimens.

### Paraberismyia tzontehuitza

Woodley, 1995

http://species-id.net/wiki/Paraberismyia_tzontehuitza

Figs 6 , 25 – 33

Paraberismyia tzontehuitza Woodley, 1995: 136.

#### Diagnosis.

*Paraberismyia tzontehuitza* can be distinguished from other species in the genus by having the combination of cell *cu*p completely covered with microtrichia and the anepisternum with a bare, shiny medial area. The other species with cell *cu*p completely covered with microtrichia, *Paraberismyia mathisi*, has the anepisternum completely covered with tomentum. Also, the female of *Paraberismyia tzontehuitza* lacks the tomentose spots along the lateral margins of the upper frons (Fig. 6) that are present in *Paraberismyia mathisi*.

#### Redescription.

**Figures 25–26.**
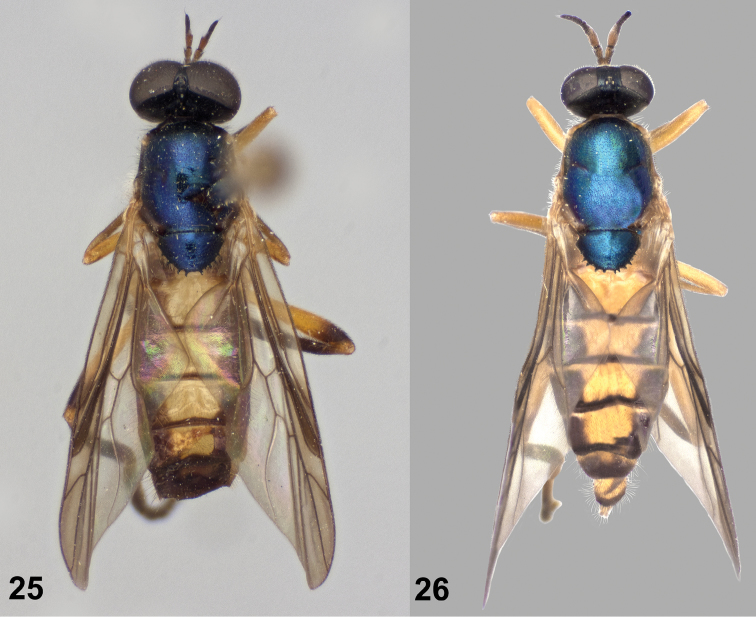
Dorsal views of *Paraberismyia tzontehuitza* Woodley. **25** Male (holotype) **26** Female (paratype).

**Figures 27–28.**
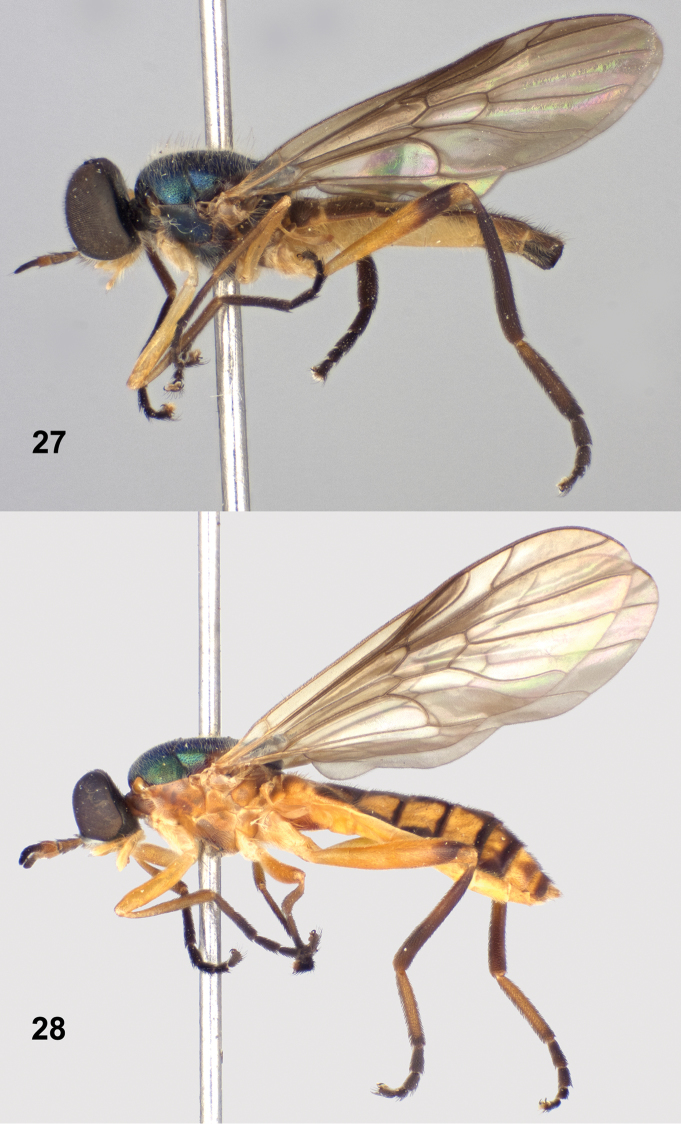
Left lateral views of *Paraberismyia tzontehuitza* Woodley. **27** Male (holotype) **28** Female (paratype).

**Male** (Figs 25, 27). *Head*: Black, without metallic reflections; lower frons and face densely grayish white tomentose, occiput also tomentose except for median occipital sclerite, but tomentum is dark, not strongly contrasting with background coloration, median area of upper frons very sparsely tomentose; upper frons and lower frons just above antennae with blackish pilosity which is shorter than the first antennal segment; face similarly pilose with somewhat longer hairs, with a few yellowish hairs intermixed; gena with pale yellowish hairs a little longer than those of face, occiput with some long pale hairs and more numerous short, inconspicuous blackish hairs; eye densely pilose, hairs brownish black, about half length of first antennal segment; antenna 1.0 times length of head, antenna with first two segments and first flagellomere yellowish, apical flagellomeres brownish black; first two antennal segments with stiff black hairs, longer hairs on flagellum black; palpus yellow, with numerous long hairs, most of which are pale yellowish, a few dark hairs present toward apex of second segment; proboscis yellow.

*Thorax*: Scutum and scutellum metallic bluish green (Fig. 25), with vague purplish reflections, lateral portion of postpronotal lobe yellowish, remainder of this and postalar callus brownish; pleura blackish brown with faint metallic reflections, but anepisternum and katepisternum more strongly metallic, not much different in coloration from mesonotum, some sutural regions below wing yellowish; mesonotum rather finely, densely punctate; thorax with grayish white tomentum present over most of prothorax, anterior margin of anepisternum, entire katepimeron and meron, anatergite, mediotergite, and subscutellum; anepisternum on dorsal half bare and shiny medially; mostly pilose with long, erect pale hairs, a little longer than first two antennal segments combined, intermixed with short pale hairs on scutum, with middle of anepisternum, entire katepimeron, meron, anatergite, mediotergite, and subscutellum bare; hind tarsus with first three tarsomeres slightly inflated; legs (Fig. 27) yellowish, except front and middle tibiae are brownish beyond basal third, especially distinct on front leg and a broad ring near apex of hind femur, hind tibia except extreme base, and all tarsi are brownish-black, with basitarsi slightly paler near bases; legs short pilose, posterior surfaces of all femora with longer hairs, posteroventral surface of hind tibia with a few longer hairs, coloration of pilosity similar to cuticular ground color, except apices of femora have blackish hairs; wing hyaline with vague brownish infuscation, especially noticeable in cells br, r_2+3_, r_4_, r_5_, and widely along veins, cell r_1_ brown, veins brownish, yellowish at extreme base of wing; cell *cu*p with entire surface covered with microtrichia; halter yellowish, vaguely suffused with brownish.

*Abdomen*: Tergites (Fig. 25) dark brownish, except medial third of each pale, translucent yellowish, but brownish on each segment beyond tergal groove, the pale area slightly wider anteriorly on segments 2–5, with very narrow extension toward lateral margin, reaching lateral margin only between first and second segments; sternites more extensively yellowish, with small, vague brownish spots on segments 2–5, more brownish beyond segment 5; tergites vaguely, almost imperceptibly tomentose, quite shiny, with short, rather sparse blackish hairs medially, becoming quite long laterally, with a few pale hairs laterally which are about length of antennal flagellum; sternites with short, yellowish hairs.

**Figures 29–33.**
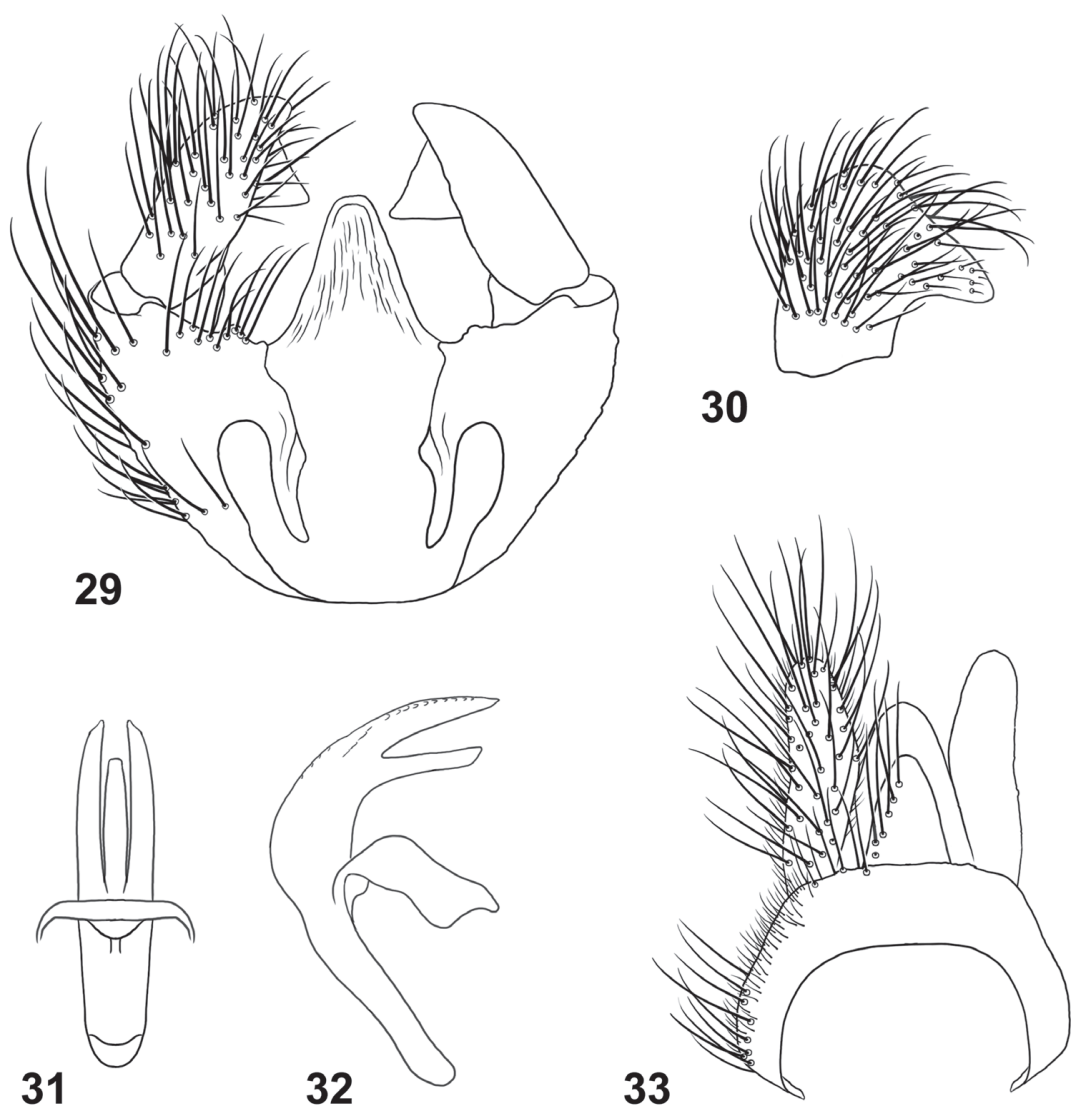
Male terminalia of *Paraberismyia tzontehuitza* Woodley. **29** Genital capsule, dorsal view **30** Gonostylus, lateral view **31** Phallic complex, dorsal view **32** Phallic complex, left lateral view **33** Epandrium and postgenital segments.

*Terminalia*: Gonocoxites (Fig. 29) with lateral margins tapering anteriorly, slightly arcuate, with low, broadly rounded process ventral to gonostylus; gonocoxal apodemes short, not reaching anterior margin of genital capsule; synsternite of genital capsule with triangular-shaped process that is narrowly rounded at apex (Fig. 29); gonostylus (Figs 29, 30) slightly arcuate, with internal triangular process near apex of ventral margin; phallic complex trifid (Figs 31, 32), strongly arcuate in lateral view, lateral lobes slightly arcuate medially, medial lobe distinctly shorter than lateral lobes; epandrium (Fig. 33) narrow, posterior margin weakly rounded; cercus narrow, very slightly arcuate laterally, apex narrowly rounded.

*Length*: 8.1 mm.

**Female** (Figs 26, 28). Differs from male as follows: *Head*: Frons 0.22–0.25 width of head at anterior ocellus; upper frons (Fig. 6) black, without metallic reflections, very finely punctate; tomentum similar to that of male, but occiput slightly paler, and lower frons has a medial, rounded bare area which extends from upper frons; pilosity of head shorter, at most one-half length of first antennal segment, except on gena; upper frons evenly, sparsely pilose with brownish black hairs; antenna longer than in male, 1.3–1.5 times length of head, flagellomeres less compact, second and internal surface of third flagellomeres yellowish; palpus more robust.

*Thorax*: Scutum and scutellum metallic bluish green (Fig. 26), but postpronotal lobe, postalar callus, and entire remainder of thorax yellowish (Fig. 28), except subscutellum and mediotergite, which are brownish; tomentum as in male, but more whitish on pale cuticular areas; pilosity generally shorter, on scutum at most as long as first antennal segment; hind tarsus without inflated tarsomeres; leg coloration as in male (Fig. 28), but front and middle tibiae are wholly yellow, hind femur with a brownish black blotch near apex which is less extensive than in male, hind tibia brownish, not as dark as in male, front and middle basitarsi yellowish on basal halves, hind basitarsus yellow except at apex; pilosity of legs similar to that of male, but posterior hairs on femora are shorter, hairs of hind basitarsus and front and middle tibiae dark, despite yellow cuticular coloration; halter yellowish.

*Abdomen*: Tergites (Fig. 26) with yellowish areas not especially translucent, first tergite mostly yellow, tergites 2–5 with yellow narrowly reaching lateral margin at bases, tergite 6 with medial yellow spot on anterior half, tergite 7 with vague lateral yellow spots; sternites entirely yellow; cerci with first segment longer than the short-ovoid second segment, first segment yellow, second slightly infused with brownish coloration, hairs pale on first segment, mostly dark on second.

*Length*: 6.3–8.3 mm.

#### Distribution.

Known only from the state of Chiapas, Mexico.

#### Type material.

I have re-examined the male holotype and 6 female paratypes (all CNC) originally cited by Woodley (1995: 137). The specimens have slightly differing elevations cited on their labels ranging from 9400 feet (2865 m) to 9600 feet (2927 m) from the locality: **MEXICO:** Chiapas, Mt. Tzontehuitz, 16°50'N, 92°35'W. The elevations cited indicate a locality near the summit of the peak

#### Additional material.

One male (USNM), **MEXICO:** Chiapas, El Triunfo (49 km S of Jaltenango, 15°39.4'N, 92°48.5'W), 1300–2000 meters, 13–15.v.1985, W.N. Mathis.

#### Etymology.

The species epithet, *tzontehuitza* (Woodley 1995: 136), is a noun in apposition referring to the type locality.

#### Remarks.

The male specimen from El Triunfo has the hind femur a little more extensively darkened apically, and the knob of the halter is brownish. The male genitalia are identical to the holotype maleso these slight differences are attributable to infraspecific variation.

## Supplementary Material

XML Treatment for Paraberismyia

XML Treatment for Paraberismyia chiapas

XML Treatment for Paraberismyia mathisi

XML Treatment for Paraberismyia triunfo

XML Treatment for Paraberismyia tzontehuitza
